# The consequences of face-threatening feedback on innovators’ psychological safety, affect, and willingness to engage in future innovation projects

**DOI:** 10.3389/fpsyg.2023.1060617

**Published:** 2023-01-26

**Authors:** John A. Daly, Alf Steinar Sætre

**Affiliations:** ^1^Departments of Communication Studies and Management, University of Texas-Austin, Austin, TX, United States; ^2^Department of Industrial Economics and Technology Management, Norwegian University of Science and Technology, Trondheim, Norway

**Keywords:** politeness theory, face sensitive messages, face threatening messages, termination messages, psychological safety, willingness to innovate

## Abstract

While there is substantial research on how firms successfully end project initiatives deemed unsuitable for them very few studies focus on how leaders and managers communicate termination messages. Drawing from politeness theory and organizational support theory we explore the impact termination messages varying in face sensitivity have on innovators’ feelings of psychological safety, affect, and their willingness to continue to innovate We find that face-threatening messages significantly and negatively affect innovators’ psychological safety, affect, and willingness to further innovate. The negative effects are amplified when innovators feel high commitment to their projects.

## Introduction

In this article we examine how people respond when projects they are involved with are terminated. More specifically, we examine how termination messages are communicated and people’s perceived commitment to terminated projects affect their emotional responses.

Extensive research has been conducted on the management of innovations over the last few decades. In that literature, significant attention is paid to topics such as how organizations encourage innovations, how innovations emerge, how they reach markets, and how innovators successfully generate commitment from organizational leaders for their initiatives (Kanter, 2020; Bingham and McDonald, 2022). The presumption of much of this research is that new ideas are, definitionally, good ones. Yet, clearly, this is not always true. History is dotted with examples of ideas that were successfully developed within organizations only to fail in the marketplace (e.g., Google glasses, Tata’s Nano, Microsoft’s Zune). The consequences of sticking with weak ideas are potentially immense—not simply the loss of money, but missed opportunities to explore other ideas, reputational costs, as well as the demotivating effects on employees seeing ideas they are involved in falter.

Why ideas that should be terminated early-on get to market is an interesting question. There are numerous explanations in the research (see Table 1). Given the difficulty of terminating projects within organizations, some work has explored how organizations, and their leaders and managers, successfully stop initiatives. Terminating a project is “a dynamic advocacy process that unfolds over time and is influenced by performance judgments and performance thresholds” (Green et al., 2003, p. 419). Some projects are terminated quickly while others fester and even resurrect again and again. A common strategy organization use to terminate ideas is the use of formal methods such as decision-gate processes where decision-makers, using established criteria, assess proposals (Cooper, 1993). Research has identified a variety of other ways firms may terminate initiatives (see Table 2).

**Table 1:** Explanations for continuing investments in bad project intiatives.

• Escalation of commitment (sunk hole; Sleesman et al., 2018)
• People’s tendency to attribute failures to external causes (Myers et al., 2014)
• Personal closeness to the initiative (Conlon and Garland, 1993)
• Managerial advocacy for the project (Lechler and Thomas, 2015)
• Unrealistic optimism (Huang et al., 2019)
• Hope and team engagement (Denicol et al., 2020)
• “Trendiness” of the initiative (Green et al., 2003)
• Overconfidence (Markovitch et al., 2015)
• Sense of personal responsibility for the project (Schmidt and Calantone, 1998)
• High levels of slack (George, 2005)
• Negative personal consequences of admitting failure [e.g., stigmatization, lower rewards, less important assignments (Cannon and Edmondson, 2001)]
• Contextual variables such as economics (Guiso et al., 2013)
• Thought-worlds (Weeth et al., 2020)
• Anticipated regret involved in shutting projects down (Sarangee et al., 2013)

**Table 2:** Research-based techniques for terminating initiatives.

• Using third party assessments and visual displays of information (Behrens and Ernst, 2014)
• Creating pre-decisional accountability (Moser et al., 2013)
• Changing strategic focus (Krishnan and Ulrich, 2001)
• Eliciting affirmations of important values (Sivanathan et al., 2008)
• Tracking projects on pre-established standards, accountability (Simonson and Staw, 1992; Kirby and Davis, 1998)
• Understanding manager’s rational or intuitive gatekeeping preferences (Eliëns et al., 2018),
• Conducting post-mortems of projects to learn, for the future, what should and should not be done, making changes in the reward system [e.g., from project completion to project success, rewarding failure (Copley and Hirschler, 2014)],
• Sponsoring “bake-offs” between competing projects, engaging independent testing, and de-escalating feature-level components (Sarangee et al., 2014).

One concern that arises when projects are terminated is the impact of those decisions on the people involved. Project terminations can be emotionally distressful (Kibler et al., 2021; Schaubroeck et al., 2021). Done poorly, terminations may sap innovators’ creativity and their willingness to learn and innovate in the future (Shepherd et al., 2011) as well as their desire to stay in their organization (Ng et al., 2022). In a recent survey, 85 percent of executives polled said that fear holds back innovation efforts often or always in their organizations. When probed, three sorts of fear affected the willingness of employees to innovate: fear of criticism, fear of uncertainty, and fear of negative impact on one’s career (Furstenthal et al., 2022)—all of which can emerge after a prior project has been terminated.

In this project we examine how the ways in which project termination messages are communicated affect people’s sense of well-being and their feelings of psychological safety (Edmondson, 1999). Few studies directly address message strategies related to termination. Smith et al. (2021), for example, looked at how failure was enacted by language choices such as metaphors, narratives, jargon, and catch-phrases. Daly et al. (2012) suggested conceptually that managers must balance termination moves with accommodations if their goal is to encourage innovators to return with new ideas after a current one is terminated. They identified seven common strategies decision-makers use when communicating decisions to end initiatives: direct statements about termination, discussion of criteria (e.g., timing, third party evaluations, resources), punishing and demeaning through communication, discussing alternatives (e.g., testing, status quo, modifications), discouragement tied to reorganizations (e.g., spin-outs, reassignments, removing vital talent, passing decision-making on to higher level leaders, leadership changes), passive communication messages (e.g., ignoring, delay), and conversations about the challenges of implementation. They framed these strategies in terms of potential accommodation messages managers make when communicating termination including openness, respect, and education.

Our research draws, conceptually, from politeness theory. “We suggest a crucial communication construct in innovation is the degree to which termination messages “step” on the innovator’s “face.” Face is “the public self-image that every [person] wants to claim for themselves” (Brown and Levinson, 1978, p. 66.). One goal of any social interaction is to avoid stepping on another’s face. Speakers avoid imposing on, and threatening, autonomy (protecting negative face) and/or highlight common interests and assure others they are respected while avoiding overt disrespect, criticism, or disapproval (protecting positive face). Critical comments, like those possibly communicated in termination conversations, may threaten both negative and positive face simultaneously. Research finds that supervisors’ face-sensitivity affects the sense of psychological safety subordinates feel (Tynan, 2005). Unmitigated face-threats have negative emotional consequences on recipients (Cupach and Carson, 2002), on negotiation outcomes (White et al., 2004), and on judgments of the people communicating the threat (Trees et al., 2009; Jenkins and Dragojevic, 2013).

Applied to innovations, Mähring et al. (2008), in a descriptive study of project de-escalation, suggest that a critical phase in implementing an exit strategy is selling the idea to terminate to the actors involved in the project “in a way where impressions are managed so as to allow face-saving…” (p. 464). Reflecting this is Albrecht and Hall’s (1991) finding that the greater the potential threat to a person’s face, the lower the likelihood of that person engaging in talk about new ideas. King et al. (2019) demonstrated, as well, that when feedback about an innovation is delivered insensitively, future suggestions for ideas are muted. The negative emotions that may be engendered by face-threatening termination messages may reduce people’s affective commitment as well as their creativity, curiosity and willingness to explore broad opportunities (Shepherd et al., 2014; Kim and Kim, 2020). Piezunka and Dahlander (2019) found that innovators were more likely to suggest new ideas if they received an explanation (face-sensitive) for why a previous idea was rejected especially when the explanation matched the linguistic style of the prior idea.

A second broader theoretic approach we draw from is organizational support theory (Kurtessis et al., 2017). The theory argues that employees’ perceptions of how much they are valued and supported by their organization (POS) affects their work-related behaviors including innovation (Le and Lei, 2019). When employees feel well supported they reciprocate those feelings through stronger work performance and greater commitment. Integrating politeness theory and organizational support theory leads us to believe that face-threatening feedback about project termination will have negative emotional consequences on recipients which, in term, should reduce their sense of psychological safety (Edmondson, 1999) and willingness to innovate (Belschak and Hartog, 2009).

### Face-saving and psychological safety

In this study, we extend research on project termination and the application of politeness theory to applied settings by suggesting that when projects are terminated in more face-sensitive ways, people should perceive greater psychological safety than when terminated in face-threatening ways. The construct of psychological safety taps people’s perceptions of the consequences of taking interpersonal risks in the workplace. Psychological safety is related to positive organizational climates, more job involvement, greater effort, and better performance (Brown and Leigh, 1996), stronger financial outcomes (Baer and Frese, 2003), and learning (Nembhard and Tucker, 2011). Schein (1993) suggested that psychological safety insulates people from being anxious or defensive when their expectations and hopes are challenged. Earlier, Kahn (1990) described psychological safety as arising when someone is “able to show and employ one’s self without fear of negative consequences to self-image, status, or career” (p. 708). When people feel psychologically safe they are more engaged in their work and more willing to raise issues (Detert and Burris, 2007), especially prohibitive (negative) ones (Liang et al., 2012). Important to the current project, psychological safety is positively correlated with innovation-related variables such as creativity (Frazier et al., 2017), less fear of failure (Carmeli and Gittell, 2009), more initiatives (Nienaber et al., 2015), improved performance in research and development settings (Chandrasekaran and Mishra, 2012), as well as team and firm learning and innovativeness (Frazier et al., 2017).

Why might face-threatening messages lead people to feel less psychologically safe? There are numerous possible reasons including an increase in negative emotions which may anchor the rejection and consequently provoke feelings of less safety (Cooper et al., 2022), guilt (Wang et al., 2021), uncertainty (Edmondson and Lei, 2014), exhaustion (Ramarajan et al., 2008), fear of negative consequences, lower status (Wijayanto et al., 2017), increased anxiety (Porath et al., 2010), decreases in the presumed quality of the relationship between the innovator and their managers (Carmeli and Gittell, 2009), and fear of community rejection (Culpeper, 2011). All of these variables suggest the following hypothesis:

*H1*: Compared to face-sensitive messages, face-threatening termination messages lead to lower psychological safety.

### The role of project commitment

Commitment is the strength of an individual’s identification with, and involvement in, initiatives (Porter et al., 1974). Commitment is correlated with lower turnover, better team performance, trust, cooperativeness, and psychological safety (Buvik and Tvedt, 2016). In the innovation literature commitment has been conceptualized a number of ways including individual and team project commitment (Ehrhardt et al., 2014), product identification (Park and Suzuki, 2021), goal commitment (Hoegl and Parboteeah, 2006), time commitment to projects (Brown and Leigh, 1996), as well as commitment to technical innovation (Bettencourt et al., 2017). In innovation settings, project commitment is positively associated with inter-team coordination, overall performance, market success, and adherence to schedules [especially among low-interdependency teams; Hoegl et al., 2004; although this may be true only for highly innovative tasks (Hoegl and Parboteeah, 2006)].

Conceptually, both dissonance theory and investment theory (Festinger, 1957; Rusbult et al., 2012) suggest that people’s commitment to a relationship, technology, job, or ideology is inversely associated with the distress they feel when that relationship, technology, job, or ideology is ended or changed [Fine and Sacher, 1997; Jermias, 2001; Van Dam, 2005; especially when they sense unfairness in the decision (Brockner et al., 1992; Franke et al., 2013)]. Thus, we hypothesize that:

*H2*: Compared to people with little commitment to a project that is terminated, people who are highly committed to a terminated project will feel less psychologically safe.

Further, the effects of the way in which project termination is communicated may be exacerbated when people have strong commitment to their project. We suspected that when highly committed people are told in face-threatening ways that their projects are being terminated, their sense of psychological safety should be lower than in other conditions. The impact of commitment on the relationship between face-threatening messages and psychological safety can also be conceptually tied to social exchange theories. Greater commitment to a project represents a greater investment and when that investment is dismissed, injustice (distributive, procedural, and interactional) may be perceived that amplifies greater psychological distress (Piccoli and De Witte, 2015) and less psychological safety. Thus, we hypothesize:

*H3*: People who are highly committed to their project and experience a face-threatening termination message will report significantly lower psychological safety compared to people with less commitment or those receiving more face-sensitive messages.

### Affect and willingness to innovate

In addition to psychological safety we examined the impact of face and commitment on two variables relevant to innovativeness that may be affected by how termination messages are communicated. The first was participants’ affect after receiving a termination message. The second was the willingness of participants to attempt further innovations after receiving the messages terminating their projects.

There is a substantial body of literature that demonstrates a significant relationship between affect and various indicators of creativity such as cognitive flexibility, fluency, and originality (Baas et al., 2008). We suspect that a face-threatening message would lead to less positive affect on the recipient’s part. Why? One explanation lies in affect reciprocity (Gottman, 1994) which refers to the tendency of people in relationships to reciprocate emotional responses (Salazar, 2015). A face-threatening response (a negative reaction to a project initiative) would, conceptually, engender reciprocal negative affect from the recipient while a more face-supportive response should lead to more positive affect. Research, for example, has suggested that failure experiences impact affect-related variables such as rumination (Schaubroeck et al., 2021). Thus:

*H4*: Compared to face sensitive messages, face-threatening messages will lead recipients to feel less positive affect.

The concept of willingness to innovate has been approached from institutional and economic perspectives (e.g., Chrisman et al., 2015; Corchuelo and Mesías, 2015; Pellegrino and Savona, 2017), studies of innovation leadership (e.g., Hill et al., 2014), and as an individual difference (e.g., Hurt et al., 1977; Gibson and Dembo, 1984). More relevant to our project, work on entrepreneurial orientation has long included, conceptually, the notion of willingness to innovate. Connecting entrepreneurial orientation with project failures is work by Wolfe and Shepherd (2015). While there is, as far as we know, no research focused on how the messages innovators receive about their notions affect their situational willingness to innovate in the future we suspect that face-threatening messages will discourage future innovation. We based this supposition on research on work motivation that demonstrates that negative responses to people’s work discourages those people from continuing that activity (Diefendorff et al., 2022) while face-supportive messages about a behavior encourage future similar behavior (Trad et al., 2014). Thus,

*H5*: Individuals who receive face-threatening feedback about their innovations will be less willing to engage in further innovation compared with those who receiving face sensitive feedback.

## Materials and methods

One hundred and twenty-four individuals participated in this project. Participants were graduate business students enrolled in three different programs—a traditional MBA, an executive MBA, and a MS executive program in technology commercialization (There was no effect of course on the results we describe later). The experiment was conducted using paper-and-pencil simulation during classes. Participants received no compensation for participating. All had at least 2 years of work experience, and most, far more (mean = 10 years; SD = 6.88). They worked in a variety of organizations and industries. Common industries were technology, health care, banking and investments, construction, government and education, military, and consumer products. The median age of participants was 31 and 68% were males. Participants were asked about their past innovations using three items: “I have often proposed new ideas when working in organizations,” “I pride myself on how I am able to generate new ideas,” and “I tend to have lots of new ideas at work.” Each item was followed by a seven-point scale ranging from strongly disagree to strongly agree (*alpha* = 0.94 and omega = 0.94). For the first item, a measure of experience in proposing ideas in the workplace, over 75% of the sample reported a five or greater. This was an experienced sample of respondents.

Participants were randomly assigned to one of four conditions. Each participant read a vignette that manipulated the two independent variables: face (threatening or supportive) and commitment (low or high). We approached the research using experimental vignette methods (EVM, Aguinis and Bradley, 2014). While most work reviewed in this study uses retrospective reports, there are challenges to that sort of approach to address hypotheses such as ours. EVM is a well-established technique for examining the causal effect of variables and allows greater control over, as well as independence of, the manipulated variables In line with Behrens and Patzelt (2016) we felt it was more important to clearly define specific independent variables that would, in the typical work setting be perhaps highly correlated with one another. Other scholars interested in communication strategies, the delivery of feedback as well as face have used a similar approach (e.g., Hodgins and Liebeskind, 2003; Belschak and Hartog, 2009; Ruppel, 2018; Hadden and Frisby, 2019; King et al., 2019; Shepherd et al., 2019; Bolino et al., 2022; Ellis, 2022). We followed the best practices described by Aguinis and Bradley (2014).

Participants read one of four vignettes that varied on commitment and face. The high commitment vignette said:

Imagine you have been deeply engaged with a small team working on an idea for the last twelve months. You have worked very hard on this. It is your idea and you have become a spokesperson for it. There has been some managerial resistance to the study already.

While the low commitment vignette said:

Imagine that you have been intermittently working with a small team on an idea for the last few days. You have not worked very hard on this. Even though it isn’t your idea, you’ve become a spokesperson for it. There has been some managerial resistance to the idea already.

The commitment manipulations were drawn from research associating commitment with both effort and psychological ownership (Sauermann and Cohen, 2010; Montani et al., 2017; Mustafa et al., 2022)

What followed was the face manipulation. We initially operationalized the face conditions based upon Daly et al. (2012) framework highlighting moves that were seen as especially face-threatening and face-sensitive as well as prior research (Jenkins and Dragojevic, 2013) on forceful language (controlling and demeaning language choices). After generating preliminary versions, we discussed the notion of face and innovation with a group of 10 experienced new product managers in the fields of energy, IT, and transportation. We asked them to generate examples of how face-threatening and face-sensitive exchanges might happen in their firms. (We were somewhat surprised by how negative some of the descriptions of face-threatening messages were. We asked the managers who provided them whether they had ever actually heard such messages. They assured us that there were certainly some people they work with, and for, who say far worse than what they described. “Engineers have very limited social skills.” “Some technology managers just get right up in your face and scream at you.”). Further, we drew from research on abusive supervision where descriptions are often face-threatening in ways similar to the one we used. For example Yu and Duffy (2021) used a scenario in one study that included “ridiculing a subordinate, telling a subordinate his or her thoughts are stupid, telling a subordinate he or she is incompetent, or being rude to the subordinate. And in their experimental manipulation in a second study” the ‘supervisor’ entered the lab displaying abrupt, loud, impolite, and inpatient demeanor while delivering negative and belittling feedback (“Your evaluation provides too little value to help me make the final decision,” You have no idea what a good candidate looks like,” “A 10-year old could do a better job!”). From thee descriptions we heard from product managers and drawing from work on abusive supervision we revised the preliminary descriptions to create the descriptions we used in this study. The face-threatening description read:

Your boss has been teasing and humiliating you in meetings about your idea. He has also attacked your motivation for pursuing the idea and has regularly suggested that if you pursue the idea there could be negative consequences for your career. Yesterday you came in to explain your project to him. He paid little attention as you explained your project. This morning he calls you into his office and says the project is going to be terminated. He provides no real feedback about the business and technical reasons for ending the project. He tells you to stop thinking about the project and tells you that he will assign you to your next project.

The face sensitive description read:

Your boss has been interested and responsive in meetings about your idea. He has admired you for pursuing the idea and has regularly suggested that there are possible positive consequence for your career if you continue to work on the idea. Yesterday you came in to explain the project. This morning he calls you into his office and says that the project is going to be terminated. He provides detailed feedback about the business and technical reasons for ending the project. He encourages you to continue thinking about new ideas and tells you that you can choose your next project.

In the hierarchy of facework strategies drawn from politeness theory, the face-threatening description would be labeled an on-record “*bald-on-record*” with no mitigation (Carson and Cupach, 2000; Limberg, 2009) while the face-sensitive statement would be considered both “*positive politeness*” and “*negative politeness*” (Brown and Levinson, 1978).

Participants then completed two manipulations checks. One was a one-item measure of commitment (“Based on what you read, how committed to the project were you prior to getting the feedback” using a seven-point scale ranging from “very uncommitted” to “very committed”). Then participants responded to a second question “Based on what you read, the response you got from your leadership was:” followed by two seven-step scales: “very insensitive” to “very sensitive” and “very negative” to “very positive.” Responses to these two scales were combined (*r* = 0.73, *p* < 0.0001).

These manipulation checks were followed by a series of measures tapping the variables we focus on in this study

### Psychological safety

We adapted Edmondson’s (1999) scale to focuses on personal reactions. Participants read a prompt that said, “After the conversation where my idea was rejected, I would feel….” followed by seven items each of which was followed by a seven-step scale ranging from “very unlikely” to “very likely.” The seven items included statements such as “That if I made a mistake it would be held against me by people in my organization,” (recoded) “That it is safe to take risks,” and “Certain that no one would deliberately act in ways that undermine my efforts.” Reliability for the seven items was *alpha* = 0.85 and omega = 0.86. A higher score implied greater psychological safety.

### Positive affect

We assessed participants’ moods by asking them “After getting the response from your leadership you feel:” followed by a series of seven-step items: sad-happy, bad-well, discontented-contented, tense-relaxed, angry-not angry, encouraged-frustrated (recoded), and pleased-miffed (recoded). These items represented hedonic toned and tense arousal items that focused on promotion aspects (Matthews et al., 1990; Baas et al., 2008). Reliability for the measure was *alpha* = 0.90 and omega = 0.92. A higher score implied more positive affect.

### Willingness to innovate

We adapted a measure of innovation created by Scott and Bruce (1994). The measure was composed of three seven-step items tapping the willingness of people to generate new ideas. The overall *alpha* for the measure was 0.86 and the omega value was 0.92. A higher score implied greater willingness to innovate in the future.

Although the three dependent measures (psychological safety, affect, willingness to innovate) are conceptually different, we conducted some confirmatory factor analyses to empirically demonstrate that they were three distinctive constructs. We calculated a single-factor (all items on one factor) and a three-factor model (items for each measure on a different factor) and calculated a chi-square differences test to see which model provided a better fit. The three-factor model [χ^2^(116) = 286.17, *p* < 0.001, RMSEA = 0.11; TLI = 0.80, CFI = 0.84] offered a significantly better fit (Δχ^2^ = 397.7, *p* < 0.001) than the single factor model [χ^2^(119) = 683.90, *p* < 0.001: RMSEA = 0.20; TLI = 0.36; CFI = 0.50]. All items loaded on their appropriate factors.

## Results

The manipulation checks revealed that participants saw the high commitment condition (M = 6.20; sd = 1.32) significantly different (in the expected direction) from the low commitment condition [M = 3.94; sd = 1.83; *F*(1,122) = 62.79, *p* < 0.0001, eta^2^ = 0.35] as well as seeing the face-sensitive condition (M = 4.86; sd = 1.40) as significantly different (in the expected direction) from the face-threatening condition [M = 1.91; sd = 1.24; *F*(1,119) = 190.71, *p* < 0.0001, eta^2^ = 0.56].

A two-way multivariate analysis of variance was conducted with face and commitment serving as independent variables and psychological safety, mood, and willingness to innovate as dependent measures. There was a significant main effect for face [*F*(3,109) = 16.53, *p* < 0.0001, Wilks’ lambda = 0.68, partial eta^2^ = 0.31] as well as commitment [*F*(3,109) = 10.24, *p* < 0.0001, Wilks’ lambda = 0.78, partial eta^2^ = 0.22]. The interaction of the two independent variables was also significant [*F*(3,109) = 3.88, *p* < 0.01, Wilks’ lambda = 0.90, partial eta^2^ = 0.10]. To better understand these results, we calculated individual two-by-two univariate analyses for each dependent measure.

### Psychological safety

There was a significant main effect [*F*(1,115) = 10.38, *p* < 0.0001, partial eta^2^ = 0.08] for face, supporting hypothesis one. Participants in the face-threatening condition (M = 23.66, sd = 9.57) reported less psychological safety than those in the face-sensitive condition (M = 29.17, sd = 8.31). There was no significant main effect for commitment [*F*(1,115) = 0.17, ns] thus not confirming hypothesis two while the interaction was significant [*F*(1,115) = 5.60, *p* < 0.02, partial eta = 0.05].^1^ The third hypothesis suggested that participants in the high commitment/face-threatening condition would feel significantly less safe than participants in other cells. A planned comparison contrasting that cell with the other three cells supported that hypothesis [*F*(3,115) = 6.03, *p* < 001]. Table 3 contains means and standard deviations. In another analysis when we controlled for self-reported innovativeness of participants (by adding innovativeness as a covariate), the results did not change.

**Table 3:** Means and standard deviations.

	Face-sensitive; low commitment	Face-sensitive; strong commitment	Face-threatening; low commitment	Face-threatening; strong commitment
Psychological safety	26.33 (9.39) *N* = 24	30.71 (7.44) *N* = 34	26.37 (9.14) *N* = 30	21.39 (9.43) *N* = 31
Affect	24.88 (7.66) *N* = 25	18.39 (5.91) *N* = 33	16.39 (5.23) *N* = 31	12.75 (4.88) *N* = 32
Willingness to innovate	15.64 (3.78) *N* = 25	15.50 (3.60) *N* = 34	11.70 (5.90) *N* = 30	10.27 (4.80) *N* = 30

### Positive affect

There was a significant main effect [*F*(1,117) = 46.56, *p* < 0.0001, partial eta^2^ = 0.28] for face confirming hypothesis four. Participants in the face-threatening condition (M = 14.56, sd = 0.5.30) reported less positive affect than those in the face-sensitive condition (M = 21.28, sd = 7.20). There was also a significant main effect for commitment [*F*(1,117) = 27.86, *p* < 0.0001, partial eta^2^ = 0.19] such that participants in the high commitment condition felt less positive affect (M = 15.37, sd = 5.81) than those in the low commitment condition (M = 20.37, sd = 7.73). The interaction was marginally significant [*F*(1, 117) = 23.36, *p* < 0.07, partial eta^2^ = 0.03]. The third hypothesis suggested that participants in the high commitment/face-threatening condition would feel significantly less positive affect than participants in other cells. A planned comparison contrasting that cell with the other three cells supported that hypothesis [*F*(3,117) = 320.45, *p* < 0.0001]. Table 3 contains means and standard deviations. In another analysis when we controlled for innovativeness (by adding innovativeness as a covariate), the results did not change.

### Willingness to innovate

**Figure 1:**
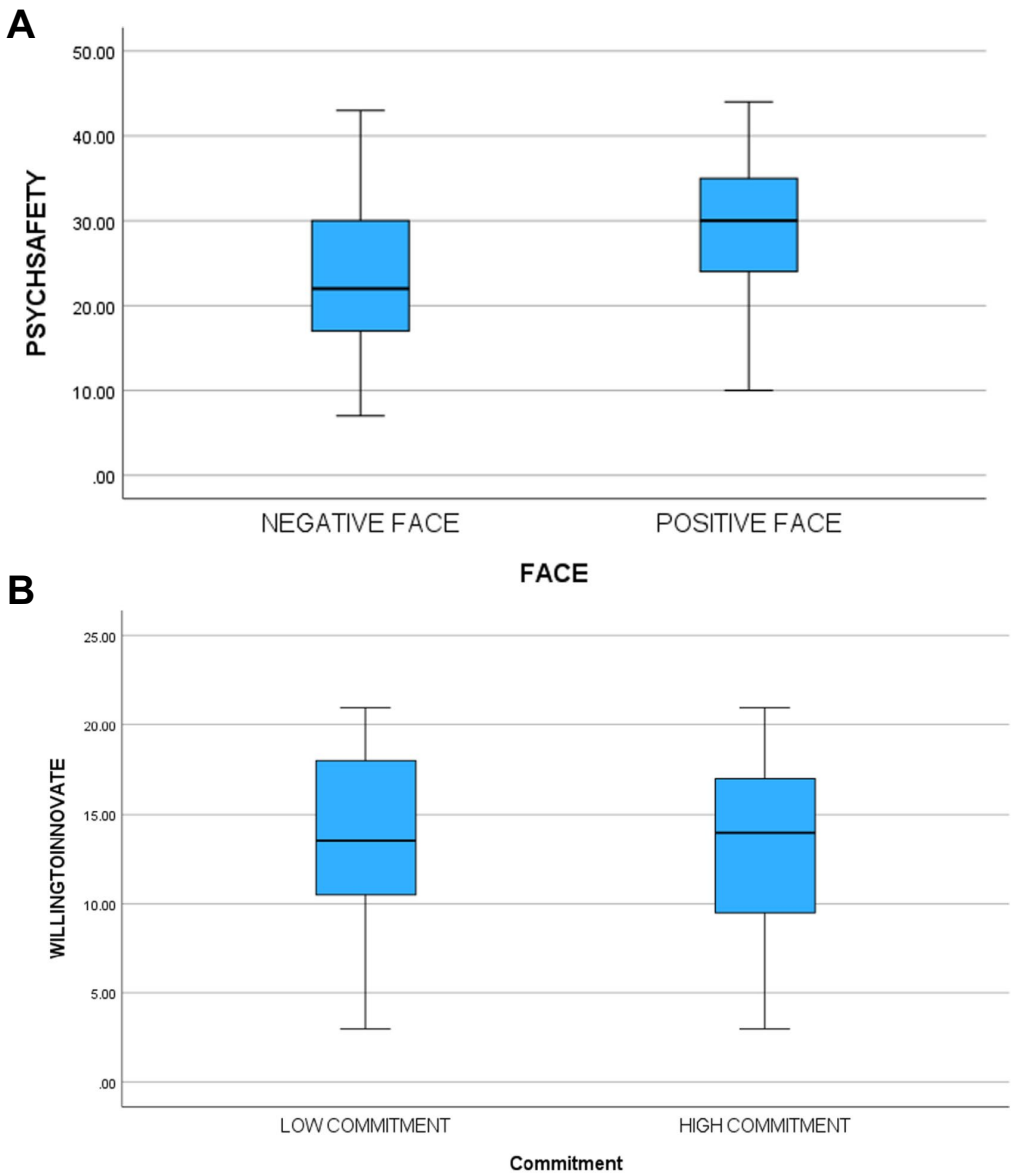
**(A)** Box plot of impact of face threat on psychological safety. **(B)** Box plot of impact of commitment on psychological safety.

**Figure 2:**
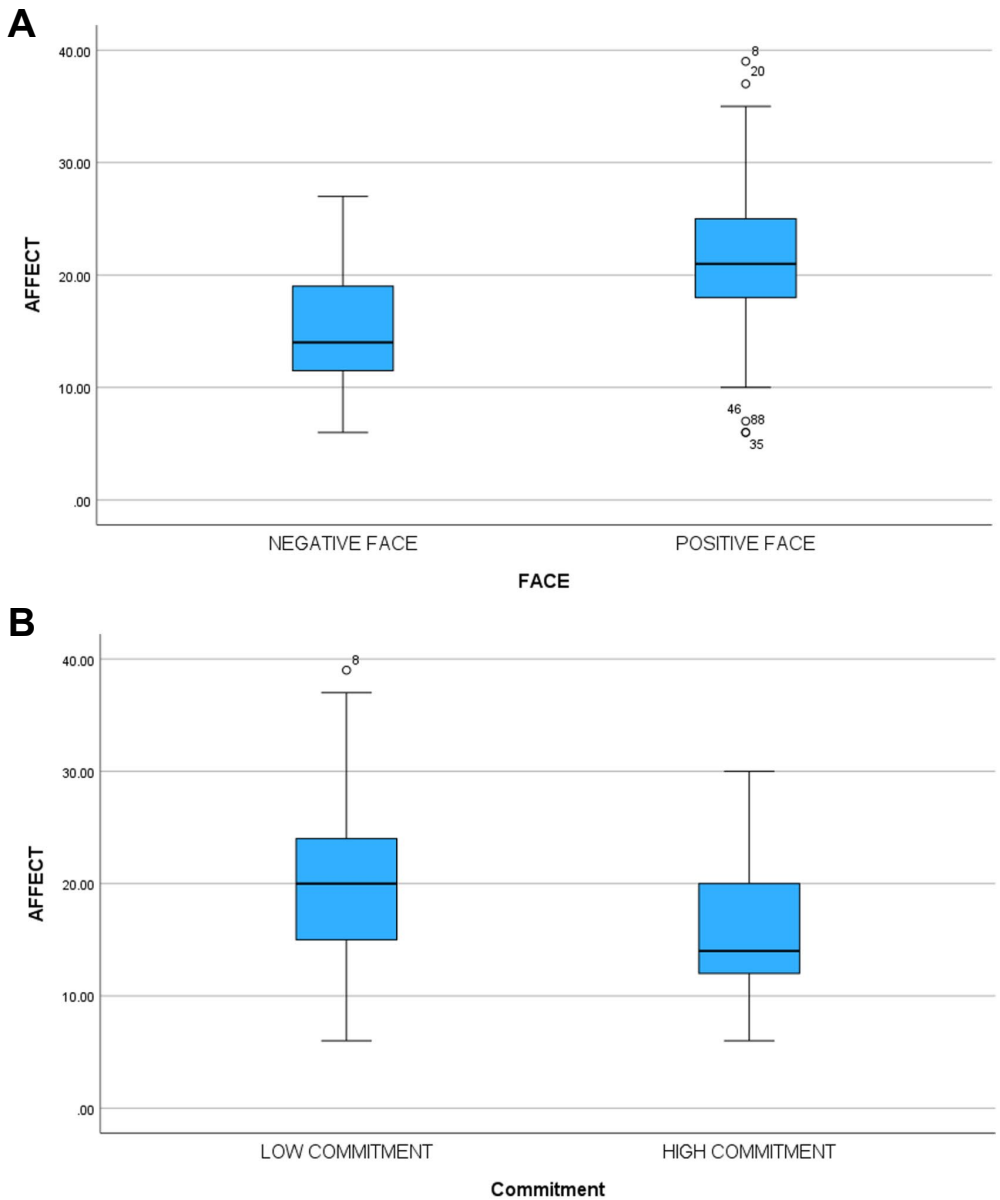
**(A)** Box plot of impact of face threat on affect. **(B)** Box plot of impact of commitment on affect.

**Figure 3:**
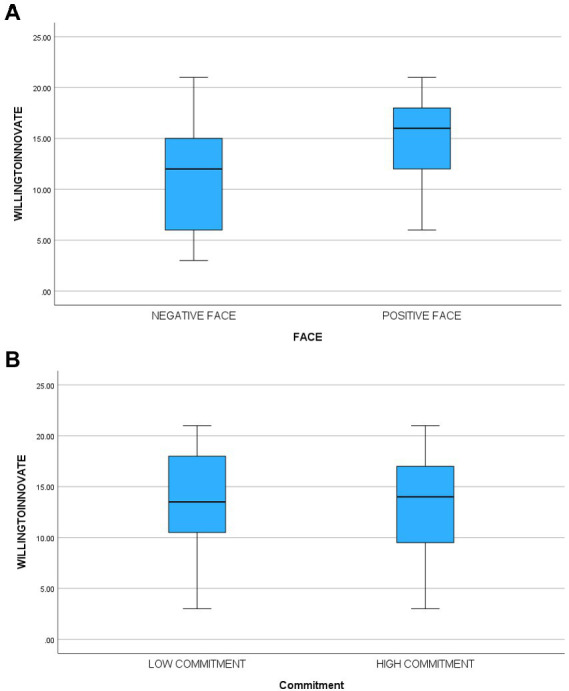
**(A)** Box plot of impact of face threat on willingness to innovate. **(B)** Box plot of impact of commitment on willingness to innovate.

**Figure 4:**
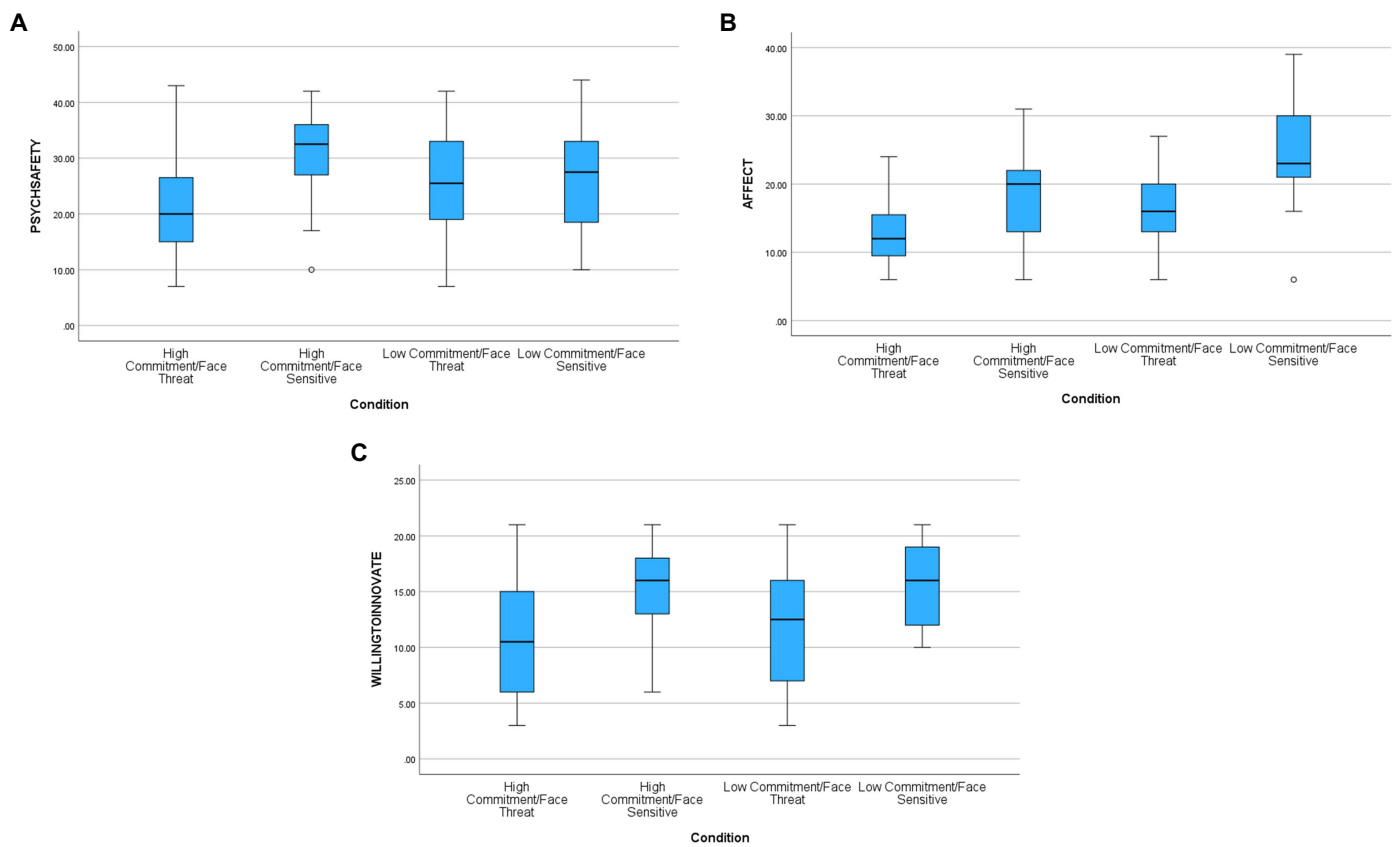
**(A)** Planned comparison for psychological safety. **(B)** Planned comparison for affect. **(C)** Planned comparison for willingness to innovate.

There was a significant main effect [*F*(1,115) = 28.56, *p* < 0.0001, partial eta^2^ = 0.20] for face supporting hypothesis five. Participants in the face-threatening condition (M = 11.05, sd = 5.36) reported less willingness to innovate than those in the face-sensitive condition (M = 15.56, sd = 3.68). There was no significant main effect for commitment [*F*(1,115) = 1.21, ns, partial eta^2^ = 0.01] nor was the interaction significant [*F*(1,115) = 0.50, ns]. The third hypothesis suggested that participants in the high commitment/face-threatening condition would report significantly less willing to innovate than participants in other cells. A planned comparison contrasting that cell with the other three cells supported that hypothesis [*F*(3,115) = 10.23, *p* < 0.0001]. Table 3 contains means and standard deviations. In another analysis when we controlled for innovativeness (by adding innovativeness as a covariate), the results did not change. Figures 1–4 below contain the box plots.

## Discussion

Drawing from both politeness theory and organizational support theory we hypothesized that exposure to a face-threatening message about project termination would negatively affect feelings of psychology safety and affect as well as decrease the willingness of people to engage in further innovation. That is what we found. Exposure to a face-threatening termination message negatively affected people’s sense of psychological safety, positive affect, and willingness to innovate in the future. These three variables are vital to innovation and risk-taking within organizations. Ideas are discretionary and when people’s innovation efforts are terminated in face-threatening ways it has negative consequences for the organization’s ability to innovate.

Innovation is definitionally risky. Not every idea is one an organization should pursue. Changing strategic needs, other innovation opportunities, lack of resources, varying risk profiles, failures to find markets, and discoveries that the ideas and numbers behind a project were wrong make it essential to sometimes terminate project initiatives. Yet, even when completely justified, the emotional costs of project termination can be meaningful to individuals invested in the project (Shepherd et al., 2009; Amankwah-Amoah et al., 2018). Done poorly, stopping an innovative project may lead to reduced affective commitment (Shepherd et al., 2011) as well as lower self-efficacy, less intrinsic motivation, and an unwillingness to again introduce new ideas (Hsu et al., 2017).

But perhaps these negative consequences can be ameliorated by how the termination decision is communicated. When attempting to successfully terminate initiatives leaders must simultaneously juggle two tasks: (a) successfully communicate the end of the project while (b) encouraging or accommodating people invested in the project to feel safe to generate future innovations (Daly et al., 2012). It is quite easy to terminate a project. At an extreme, people can simply be forced to stop an initiative. Resources necessary can be withdrawn and people involved can be threatened with the loss of their jobs were they to continue with the initiative. But terminating with encouragement is a bigger challenge. And, this is especially challenging, when, as is typical, people who create, lead, and work on projects are often highly committed to those projects.

In our study, the effects of differential commitment were mixed. We offered two hypotheses about commitment. First, that psychological safety, affect, and willingness to innovate would decrease when people felt highly committed to the terminated project. Commitment impacted, as expected, feeling of safety and affect but not willingness to innovate in the future. Our second hypothesis was that a sense of high commitment would amplify the negative effects of face-threatening messages. This is what we found. In contrast with people who imagined being less committed to their project, people who imagined strong commitment to an initiative reacted more negatively (psychological safety and affect) to project termination. In terms of effect size, commitment had a limited impact on the dependent variables compared to face. Why? Perhaps because of our operationalization of commitment. Rusbult et al. (2012) suggests three variables (alternative, satisfaction, and investment) create strong commitment. Our operationalization focused solely on investment (in terms of positivity, time, ownership, and effort) which is most closely aligned with continuance commitment (Allen and Meyer, 1996) in contrast to affective and normative commitment. And, the absence of a main effect of commitment on willingness to innovate may also be due to our measure. Commitment to innovation (a construct different from commitment to an innovation) may insulate employees from the impact of situational variables that can affect innovation attempts (Bettencourt et al., 2017). While we find that face-threatening termination messages decrease willingness to innovate, one must recognize that paradoxically the positives of greater commitment must be contrasted with the negatives of more commitment. For instance, one reason for the escalation of commitment in product development is the degree of personal responsibility innovators feel they have for their product (Schmidt and Calantone, 2002).

There are numerous avenues for future studies related to how termination messages about innovation projects are communicated. For example, certain employee characteristics may affect how termination messages are perceived and handled. Employee political skills, engineering mindset, desensitization to termination, coping self-efficacy, an intuitive cognitive style, motivation to learn, and resilience to innovation (Shepherd et al., 2009; Zhao, 2011; Mueller and Shepherd, 2016; Todt et al., 2018; De Clercq and Pereira, 2019; Morais-Storz et al., 2020) all may affect people responses to termination messages. Some people may be better able to “bounce back” from rejection (Wolfe and Shepherd, 2015). As well, team characteristics such as reflexivity (Rauter et al., 2018) as well as the approach management takes to termination (e.g., strategic vs. undisciplined, Corbett et al., 2007) might also be considered.

An interesting question is how managers who use face-threatening messages to terminate innovation-related projects might attenuate the effects and encourage future innovation. One might consider research on forgiveness (Merolla, 2014) as well as trust repair (Kramer and Lewicki, 2010) that suggests tactics such as non-defensive explanations and proportionately explicit apologies along with reparations (Hodgins and Liebeskind, 2003; Zhang et al., 2019) may reduce the impact of face-threatening messages. Politeness theory suggests that the impact of face-threatening messages may be less when the speaker has power or the situation requires a speedy and efficient response (Jenkins and Dragojevic, 2013). Speed may be relevant. Some, but not too much, delay in terminating a project may be optimal. It gives innovators a chance to reflect and emotionally prepare for the end of their projects (Shepherd et al., 2009). The personal history of innovators when it comes to idea termination may matter, as well. Deichmann and van den Ende (2014) found that when people have a few of their ideas rejected they are more likely to propose new ones. On the other hand, when many of their ideas have been rejected, they become less likely to initiate new ideas. It would be valuable to investigate to what extend the effect of face-threatening messages is moderated by “how” innovation projects are terminated.

It may also be helpful in future work to consider alternative theoretical explanations and distinctions. While politeness theory offers a useful theoretical rationale for the study’s finding one could also draw from reactance theory (Brehm and Brehm, 1981) to explain the effects of face-threatening statements. The two theories differ on explanatory mechanisms—politeness emphasizing interactional rules while reactance highlighting threats to autonomy. Future research may determine which is the better explanation for the negative effects of certain termination messages. Understanding the emotional dynamics underlying people reactions to termination messages might be framed, as well, in terms of appraisal theories of emotions (Lazarus, 2001). Research on abusive supervision has recently taken this conceptual turn (Oh and Farh, 2017) suggesting a three-stage model: (1) attribution and appraisal of the behavior, (2) experienced emotions as a function of those attributions and appraisals, and (3) behaviors associated with those emotions. In the entrepreneurship literature (Williams et al., 2020) there is evidence that people’s attributions (e.g., internal-external, controllability) for business failures affects their decisions to create other businesses. The same process probably operates immediately and over time when internal projects are terminated. After initial disappointment, how people emotional and cognitively frame the termination messages may affect the long-term consequences of those messages (Kibler et al., 2021). In the current study we focused on immediate reactions (safety, affective reactions, and willingness to innovate). Over time, though, innovators may make attributions (e.g., externalize the response; see the decision as not personally controllable) that might assuage their initial responses.

### Limitations

As with any project, there are clear limitations to this study. One is the use of an experimental model where commitment and face were manipulated orthogonally *via* descriptions. In accord with experimental vignette methods we used an experimental model to create clear and unambiguous conditions of face-supportive and face-threatening messages. Moving beyond the experimental paradigm used in this study and examining actual termination events is an important next step. In reality, managers’ messages about termination likely vary along a continuum from face-threatening to face-sensitive. If it were possible to calibrate the degree to which termination messages are face-threatening then we would be able to determine the relative impact of different sorts of messages. Research clearly demonstrates people’s bias towards attending to more negative messages (Bledow et al., 2017) so perhaps there is a non-linear relationship between the degree of face-threat and people’s reactions to termination messages (Wolfe and Shepherd, 2015). Termination messages are also seldom single statements. Instead, in typical organizational settings there are likely numerous warnings and hints about an impending decision. People, we suspect, are seldom surprised by termination messages. They may be surprised, though, by the way those messages are delivered. Do people ever actually get termination messages that might step on their face? In a brief follow-up poll we asked 89 engineers involved in R&D efforts at a major global semi-conductor company whether they had ever received face-threatening feedback about their initiatives (we described potentially face-threatening sorts of statements). Sixty four percent of them said they had personally experienced face-threatening feedback about initiatives.

A second limitation is the nature of the dependent variables. Psychological safety, affect, and a willingness to innovate further have all been tied to innovative behaviors. But our data alone does not provide clear evidence that face-threatening behaviors or low commitment decreases actual future innovative attempts. Future research will need to do that.

Another consideration are cultural differences that might affect these results. Cultures differ when it comes to raising and handling face-related issues (e.g., Zhang et al., 2019). As well, corporate culture may matter. Shepherd et al. (2011) argue that in some organizations, failure is treated as nothing special. Intel, for example, was, for many years, notorious about how direct people were with their reactions to others proposals (Johnson and Phillips, 2003) and firms like Bridgewater and Netflix pride themselves on “hard-edged and fearlessly candid” feedback (Buckingham and Goodall, 2019). In those “constructive conflict” cultures even a face-threatening statement may have little consequence (Danneels and Vestal, 2020). Similarly, the professional culture may matter. The engineering culture may find rejection, regardless of how it is phrased, less negative than some other professional cultures (Shepherd et al., 2014) especially if failure is normalized (Shepherd et al., 2011).

### Managerial implications

Projects sometimes need to be terminated. How managers communicate with people involved in the terminated project matters. Communicated well, with respect, those messages can encourage recipients to feel safe to continue innovating in the future. On the other hand, if messages “step” on the “face” of recipients, innovators will be less willing to risk future innovation projects. Successful innovation managers should carefully construct termination messages that (1) offer recipients respect for their innovative work allowing them, (2) give them reasonable explanations for the decision to end the project while (3) encouraging them to engage in future projects with autonomy. Being face-sensitive when offering project termination messages is especially important when innovators are highly committed to their projects.

## Data availability statement

The raw data supporting the conclusions of this article will be made available by the authors, without undue reservation.

## Ethics statement

The studies involving human participants were reviewed and approved by The University of Texas Human Subjects Committee. The patients/participants provided their written informed consent to participate in this study.

## Author contributions

Both authors listed have made a substantial, direct, and intellectual contribution to the work and approved it for publication.

## Conflict of interest

The authors declare that the research was conducted in the absence of any commercial or financial relationships that could be construed as a potential conflict of interest.

## Publisher’s note

All claims expressed in this article are solely those of the authors and do not necessarily represent those of their affiliated organizations, or those of the publisher, the editors and the reviewers. Any product that may be evaluated in this article, or claim that may be made by its manufacturer, is not guaranteed or endorsed by the publisher.

## Footnotes

^1^We did not calculate *a priori* the power of our tests, but in line with (Gelman and Carlin, 2014) we did compute *post hoc* indicators (S and M indices) using the “rdesigni” power analysis routine in Stata 17. **Table:**

Main effect		Type S error rate	Type M error rate
Psy Safety	Face	0.08	4.73
	Comm	0.50	2715.61
Positive Affect	Face	0.25	10.13
	Comm	0.33	15.30
Willingness to Innovate	Face	0.25	9.96
	Comm	0.48	213.12
